# Effects of Prenatal Exposure to Alcohol and Smoking on Fetal Heart Rate and Movement Regulation

**DOI:** 10.3389/fphys.2021.594605

**Published:** 2021-07-30

**Authors:** Maristella Lucchini, Lauren C. Shuffrey, J. David Nugent, Nicoló Pini, Ayesha Sania, Margaret Shair, Lucy Brink, Carlie du Plessis, Hein J. Odendaal, Morgan E. Nelson, Christa Friedrich, Jyoti Angal, Amy J. Elliott, Coen A. Groenewald, Larry T. Burd, Michael M. Myers, William P. Fifer

**Affiliations:** University of Texas Medical Branch; DM-STAT, Inc.; Children’s Hospital Boston; Sanford Research; Stellenbosch University; Columbia University; ^1^Department of Psychiatry, Columbia University Irving Medical Center, New York, NY, United States; ^2^Division of Developmental Neuroscience, New York State Psychiatric Institute, New York, NY, United States; ^3^Department of Obstetrics and Gynecology, Faculty of Medicine and Health Science, Stellenbosch University, Cape Town, South Africa; ^4^Center for Pediatric and Community Research, Avera Research Institute, Sioux Falls, SD, United States; ^5^Department of Pediatrics, University of South Dakota School of Medicine, Sioux Falls, SD, United States; ^6^Department of Pediatrics, University of North Dakota Medical School, Grand Forks, ND, United States; ^7^Department of Pediatrics, Columbia University Irving Medical Center, New York, NY, United States

**Keywords:** fetal heart rate, fetal movement, autonomic nervous system, prenatal, alcohol, smoking

## Abstract

Negative associations of prenatal tobacco and alcohol exposure (PTE and PAE) on birth outcomes and childhood development have been well documented, but less is known about underlying mechanisms. A possible pathway for the adverse fetal outcomes associated with PTE and PAE is the alteration of fetal autonomic nervous system development. This study assessed PTE and PAE effects on measures of fetal autonomic regulation, as quantified by heart rate (HR), heart rate variability (SD-HR), movement, and HR-movement coupling in a population of fetuses at ≥ 34 weeks gestational age. Participants are a subset of the Safe Passage Study, a prospective cohort study that enrolled pregnant women from clinical sites in Cape Town, South Africa, and the Northern Plains region, United States. PAE was defined by six levels: no alcohol, low quit early, high quit early, low continuous, moderate continuous, and high continuous; while PTE by 4 levels: no smoking, quit early, low continuous, and moderate/high continuous. Linear regression analyses of autonomic measures were employed controlling for fetal sex, gestational age at assessment, site, maternal education, household crowding, and depression. Analyses were also stratified by sleep state (1F and 2F) and site (South Africa, *N* = 4025, Northern Plains, *N* = 2466). The final sample included 6491 maternal-fetal-dyad assessed in the third trimester [35.21 ± 1.26 (mean ± SD) weeks gestation]. PTE was associated with a decrease in mean HR in state 2F, in a dose dependent fashion, only for fetuses of mothers who continued smoking after the first trimester. In state 1F, there was a significant increase in mean HR in fetuses whose mother quit during the first trimester. This effect was driven by the Norther Plains cohort. PTE was also associated with a significant reduction in fetal movement in the most highly exposed group. In South Africa a significant increase in mean HR both for the high quit early and the high continuous group was observed. In conclusion, this investigation addresses a critical knowledge gap regarding the relationship between PTE and PAE and fetal autonomic regulation. We believe these results can contribute to elucidating mechanisms underlying risk for adverse outcomes.

## Introduction

Many deleterious effects of alcohol consumption during pregnancy on fetal development, birth outcomes, and subsequent childhood development are well-documented (Popova et al., 2016). Prenatal alcohol exposure (PAE) is associated with increased risk of preterm birth, stillbirth, low birth weight, birth defects, and risk for neurodevelopmental disorders (Bailey and Sokol, 2011). Prenatal tobacco exposure (PTE) has also been associated with higher risk of negative outcomes including preterm birth and stillbirth as well as sudden infant death syndrome (SIDS), attention deficit hyperactivity disorder (ADHD) (Huang et al., 2018), and conduct disorder in offspring (Klonoff-Cohen and Lam-Kruglick, 2001; Cnattingius, 2004; Vardavas et al., 2010). Prior research has not elucidated a safe quantity or timing of alcohol or tobacco exposure during pregnancy, thus national guidelines in most countries advise against consuming any alcohol and smoking during pregnancy (American College of Obstetricians and Gynecologitsts, 2017; International Alliance for Responsible Drinking (Iard)., 2019).

A possible marker of effects of alcohol and smoking on fetal development is autonomic nervous system (ANS) activity as assessed through measures of fetal heart rate (HR) and heart rate variability (HRV). HRV is the variation in the heart’s beat-to-beat intervals and it is regulated by the ANS, through the synergistic activity of the parasympathetic and sympathetic branches (Saul, 1990; Acharya et al., 2006). With increasing gestational age (GA), fetal HR tends to decrease while HRV increases, due to increased parasympathetic activity, maturation of central mechanisms, and more frequent occurrence of breathing movements (Cnattingius, 2004).

Studies investigating the effects of PAE on fetal HRV have obtained mixed results: some, performed during maternal intoxication, showed reduced fetal HRV (Halmesmaki and Ylikorkala, 1986; Silva et al., 1987; Schneider et al., 2008). However, two other reports found that moderate levels of acute PAE resulted in no change in fetal HR (McLeod et al., 1983; Mulder et al., 1998). In other physiological studies, McLeod and co-workers found a reduction in fetal breathing movements in response to acute alcohol exposure (McLeod et al., 1983) while Mulder et al. (1998) found no change in fetal breathing movements but observed suppressed fetal eye movements immediately following alcohol exposure. Studies investigating the effect of chronic PAE are sparse and typically retrospective. Studies reporting the effects of PTE on HR and HRV have also demonstrated mixed results. Prior studies have either reported an increase in fetal HR from acute exposure to maternal smoking (Quigley et al., 1979; Kelly et al., 1984; Péterfi et al., 2019) or no statistically significant difference in fetal HR after acute exposure to maternal smoking (Barrett et al., 1981; Goodman et al., 1984; Oncken et al., 2002). Additionally, prior studies have reported decreased HRV in response to acute maternal smoking (Eriksen et al., 1984; Goodman et al., 1984; Péterfi et al., 2019). Investigations of fetal HR and HRV in chronic maternal smokers reported decreased baseline fetal HR compared to controls and reduced HRV (Kapaya et al., 2014; Spyridou et al., 2017; Zeskind and Gingras, 2018). Chronic effects of maternal smoking have also been associated with reduced fetal breathing movements (Gennser et al., 1975), reduced fetal movement (Coppens et al., 2001), and non-reactive fetal activity-acceleration determination tests (Phelan, 1980).

In sum, research to date has primarily investigated acute effects of high levels of PAE or PTE on fetal HR or HRV. Additionally, the effects of acute and chronic maternal smoking and alcohol consumption on FHR and fetal HRV have been investigated independently of one another. This leaves a significant gap in the literature for understanding the dual effects of chronic low and moderate alcohol and tobacco use during pregnancy on function and development of fetal autonomic nervous system. The dataset analyzed in this report is a subset of the Safe Passage Study conducted by the Prenatal Alcohol and SIDS and Stillbirth (PASS) Network (Dukes et al., 2014). The aim of the study was to characterize the role of prenatal exposure to alcohol, cigarettes, and other environmental stressors on SIDS, stillbirth, and FASD. The Safe Passage Study enrolled approximately 12,000 maternal-fetal dyads in the Northern Plains (NP) of the United States (North and South Dakota) and Cape Town, South Africa (SA). Both these areas are known for high risks of SIDS, stillbirth, and FASD and have high rates of alcohol consumption and smoking during pregnancy as well as other known risk factors such as recreational drug use, or prior trauma (Bulterys, 1990; Iyasu, 2002; May et al., 2005, 2014; Popova et al., 2017). The present report focuses on reporting the effects of data driven patterns of alcohol consumption and smoking during pregnancy on measures of fetal HR, HRV movement and HR-movement coupling obtained from cardiotocographic recordings of fetuses at ≥ 34 weeks gestational age (GA).

## Materials and Methods

### Participants

From 2007 until 2015, the Safe Passage Study followed the outcomes of ∼12,000 pregnancies among women from two comprehensive clinical site (CCS), one in the Cape Town area, South Africa, and one in the Northern Plains, United States. The United States site is comprised of five clinical sites in North Dakota and South Dakota, including two sites on American Indian Reservations. In South Africa, recruitment occurred from Bishop Lavis and Belhar residential areas within Cape Town, which serve mainly the multiracial population (South African multiracial ethnic group (multiracial group), which have ancestry from more than one of the various populations inhabiting the region, including Khoisan, Bantu, European, Austronesian, and East Asian or South Asian). Screening and enrollment occurred at prenatal clinics affiliated with each CCS between 6 weeks gestation up to, but not including, delivery. Ethical approval was obtained from Stellenbosch University, Sanford Health, the Indian Health Service, and New York State Psychiatric Institute. Written informed consent to record fetal HR was part of the consent for the main study. Maternal and infant charts were abstracted to obtain demographic and relevant clinical information. We excluded participants with maternal health conditions known to affects our outcome measures (gestational diabetes, preeclampsia, hypertension), psychiatric medication use during pregnancy (SSRI’s, antidepressants, classic antipsychotics, atypical antipsychotics, mood stabilizers, stimulants, antianxiety medications, or anticonvulsants), any recreational drug use during pregnancy, multiple births, and congenital anomalies.

### Self-Reported Exposure Measures

The protocol used to obtain detailed information about quantity and timing of prenatal exposure to alcohol and smoking is presented in Dukes et al. (2017). A modified Timeline Follow-back interview was employed to collect this information. Maternal smoking information was obtained through maternal interviews by trained research staff to estimate average cigarettes smoked per week for each week of pregnancy. Interviews were performed up to 4 times during pregnancy (recruitment, 20–24 weeks GA, 28–32 weeks GA, and 34–38 weeks GA). A validation study using a subset of *N* = 108 Safe Passage Study women was performed and it indicated strong concordance between maternal report and meconium biomarkers (Himes et al., 2015). As a result of the methodology for the collection of self-report exposure information, a given participant could potentially have single or multiple segments of missing information on alcohol or smoking consumption. For this reason, we imputed missing daily exposure data using a K-Nearest Neighbor approach (Sania et al., 2020). Further information can be found in the Supplementary Material 1.

We then used clustering techniques to characterize multiple patterns of maternal drinking and smoking behaviors (Pini et al., 2019). Further information on alcohol and smoking exposure clustering can be found in the Supplementary Material 2. In the present analysis we utilized six categories of PAE (no alcohol, low quit early, high quit early, low continuous, moderate continuous, and high continuous) and a four-level PTE variable (no, quit early, low continuous, and moderate/high continuous). Depression was assessed using the Edinburgh Postnatal Depression Scale (EPDS), which has been validated for use during pregnancy, at the first study visit (Cox et al., 1987; Rubertsson et al., 2011).

### Data Acquisition and Processing

Fetal assessments were performed at 34–38 weeks gestation (Mean ± SD = 35.4 ± 1.2 weeks). Assessments were completed between 9 am and 4 pm and lasted approximately 50 min. Mothers were seated in a reclining chair or were lying supine with a 15° lateral tilt and fitted with the recording equipment. Mothers were undisturbed for the first 20 min of data collection and then answered questions on alcohol and smoking habits, recreational drug use and depression during the remaining 30 min. Fetal HR and movement data were collected using a single wide-array Doppler transducer placed on the maternal abdomen connected to a Toitu MT-320 or a MT-516 model Doppler actocardiograph (Toitu Company, Ltd., Toyko, Japan). FHR and FMOV signals were digitized at 20 Hz using a custom-built physiological data acquisition hardware and software system (DATACQ, Medelex, Inc) interfaced to a laptop computer. Specific details on the acquisition protocol can be found in previous articles (Myers et al., 2017; Shuffrey et al., 2019). Further information on data processing can be found in the Supplementary Material 3.

### Outcome Parameters

Mean HR and standard deviation (SD) of HR were computed for each epoch, using only the non–interpolated values. The median fetal movement was computed for each accepted fetal HR epoch except in cases where the fetal movement signal exceeded the range of the Toitu fetal movement amplifier or was not present. These cases comprised 2.5% of all records and were due to equipment failure or user error. In addition, the cross-correlation of fetal HR and movement (heart rate/movement coupling) and the lag (seconds) between movement and fetal HR derived from the cross-correlation function were computed for each accepted 4-min fetal HR epoch. The fetal HR and movement signals were first low-pass filtered between 0.002 and 0.05 Hz using a 400-point FIR filter. The fetal movement signal was z–transformed and the fetal HR was further processed by subtracting the mean from a local regression of 6 s and negative fetal HR values were set to zero (Dipietro et al., 2001). As a further control for artifact, each segment required a minimum covariance value of 0.5 and a lag at the maximum cross–correlation greater than –15 sec or less than 0 sec (i.e., changes in FMOV were required to precede changes in FHR) (Dipietro et al., 2001). For each recording, means of all the above variables were computed for the accepted segments, for each fetal state, including state 1F, also known as the quiet fetal behavioral state, and state 2F, also known as the active behavioral sleep state. More information about fetal behavioral sleep states in available in S.M.4.

### Statistical Analyses

Linear regression analyses were used to estimate the associations between exposure categories and HR and HRV and movement parameters. We fit separate models for HR mean, SD, and the cross-correlation of fetal HR and movement parameters as outcomes. All models included sex and gestational age at assessment as covariates (Dipietro et al., 2015). We additionally adjusted for maternal education (any primary school, some high school, completed high school, and beyond high school), household crowding index (CI: 0–25th, 25–75th, and 75–100th percentile), depression scores measured with the Edinburgh scale (considered as a continuous variable), and clinical site as potential confounders. For these additional adjustments, we accounted for missing covariate data by adding a missing indicator variable in the model. Analyses were performed for all subjects combined across both sites and were repeated after stratifying by clinical site, i.e., Northern Plains and South Africa, Cape Town. Analyses were performed using R for Windows 3.6.1.

## Results

In total, 11,929 mother-infant pairs were enrolled in the Safe Passage Study. We performed fetal HR assessments on 9240. Because of their known association with outcomes (Jansson et al., 2005; Rurak et al., 2011), we excluded 1098 for maternal conditions (gestational diabetes, hypertension, and preeclampsia) and congenital abnormalities and 1,236 for psychiatric medications and recreational drugs use. We also excluded 394 for incomplete exposure data and 21 for missing covariates (sex). Our final sample included a total of 6,491, 4,025 from SA and 2,466 from NP. Roughly half of the fetuses were males. Supplementary Figure 1 shows a study consort chart.

Mean age at enrollment was 25.8 ± 5.7 years (Mean ± SD) and most participants (95.1%) had at least some high school education, and roughly half of the participants were employed. The population was composed of individuals who self-identified as white, multiracial, American Indians/Alaska natives or Other/unknown races.

A total of 51.9% of the women drank and 42.7% smoked at some point during pregnancy. Of the smokers, 14.9%, 24.1%, and 3.8% were grouped into high/moderate, low continuous and quit early groups, respectively. For alcohol 4.6%, 9.1%, 8.6%, 5.4%, and 24.3% were grouped into high continuous, moderate continuous, low continuous, and high quit early and low quit early groups, respectively.

Fetuses were assessed on average at 35.2 (± 1.3 SD) weeks of gestation. They were successively born at 39.4 (± 1.4 SD) weeks gestational age. Table 1 contains information on maternal demographic, exposure variables, and infant characteristics, including the breakdown by study site.

**TABLE 1:** Maternal and infant demographics and prenatal exposure information.

	South Africa and Northers Plain (*N* = 6491)	South Africa (*N* = 4025)	Northers Plain (*N* = 2466)
**Maternal characteristics**			
Maternal age	25.80 ± 5.72	24.99 ± 5.89	27.11 ± 5.15
**Education**			
*Any primary school*	317 (4.9%)	289 (7.2%)	28 (1.1%)
*Some high school*	3995 (46.1%)	2638 (65.5%)	357 (14.5%)
*Complete high school*	1324 (20.4%)	915 (22.7%)	409 (16.6%)
*Beyond high school*	1849 (28.5%)	177 (4.4%)	1672 (67.8%)
**Married/Partnered living together**			
*No*	2550 (39.3%)	2087 (51.9%)	463 (18.8%)
*Yes*	3925 (60.5%)	1923 (47.8%)	2002 (81.2%)
**Employed**			
*No*	2973 (45.8%)	2323 (57.7%)	650 (26.4%)
*Yes*	2954 (45.5%)	1266 (31.5%)	1688 (68.5%)
Crowding index	1.23 ± 0.89	1.55 ± 0.89	0.71 ± 0.59
**Race**			
*American Indian or Alaska native*	689 (10.6%)	0	689 (27.9%)
*Mixed ancestry*	4010 (61.8%)	4010 (99.6%)	0
*White*	1594 (24.6%)	0	1594 (64.6%)
*Other/Unknown*	198 (3.1%)	15 (0.4%)	183 (7.4%)
**Exposures**			
Edinburgh depression scale	9.73 ± 6.44	12.57 ± 5.92	5.09 ± 4.18
**Smoking**			
*No*	3717 (57.3%)	1721 (42.8%)	1996 (80.9%)
*Quit early*	244 (3.8%)	111 (2.8%)	133 (5.4%)
*Low continuous*	1565 (24.1%)	1335 (33.2%)	230 (9.3%)
*Moderate/high continuous*	965 (14.9%)	858 (21.3%)	107 (4.3%)
**Alcohol**			
*No alcohol*	3120 (48.07%)	1881 (46.73%)	1239 (50.24%)
*Alcohol low quit early*	1575 (24.26%)	721 (17.91%)	854 (34.63%)
*Alcohol high quit early*	348 (5.36%)	161 (4.00%)	187 (7.58%)
*Alcohol low continuous*	560 (8.63%)	517 (12.84%)	43 (1.74%)
*Alcohol moderate continuous*	590 (9.09%)	472 (11.73%)	118 (4.79%)
*Alcohol high continuous*	298 (4.59%)	273 (6.79%)	25 (1.02%)
**Infant characteristics**			
GA at assessment (days)	35.21 ± 1.26	34.88 ± 7.05	35.75 ± 1.42
GA at birth (weeks)	39.39 ± 1.41	39.36 ± 1.47	39.43 ± 1.32
**Sex**			
*Male*	3221 (49.6%)	1981 (49.2%)	1240 (50.3%)
*Female*	3270 (50.4%)	2044 (50.8%)	1226 (49.7%)

Table 2 shows the cross-tabulations of the exposure groups for the overall population and by site.

**TABLE 2:** Cross Tabulation of smoking and drinking groups in the overall population, South Africa population, and Northern Plains population.

		Alcohol exposure					
		
		None	Low quit early	High quit early	Low continuous	Moderate continuous	High continuous
**Overall population**							
Smoking exposure	*None*	2029	1082	198	206	147	55
	*Quit early*	95	79	23	20	17	10
	*Low continuous*	638	277	76	226	248	100
	*Moderate/high continuous*	358	137	51	108	178	133
**South Africa**							
Smoking exposure	*None*	1007	364	58	172	75	45
	*Quit early*	41	35	7	19	8	1
	*Low continuous*	526	219	53	221	220	96
	*Moderate/high continuous*	307	103	43	105	169	131
**Northern Plains**							
Smoking exposure	*None*	1022	718	140	34	72	10
	*Quit early*	54	44	16	1	9	9
	*Low continuous*	112	58	23	5	28	4
	*Moderate/high continuous*	51	34	8	3	9	2

Tables 3, 4 show the average number of drinks and the average number of binge events per trimester and the average number of cigarettes/week per trimester. Tables 5–12 summarize results from linear regression models discussed in the next sections.

**TABLE 3:** Number of drinks and binge events by trimester per alcohol group in the overall population, South Africa population, and Northern Plains population.

	Alcohol exposure					
	
	None	Low quit early	High quit early	Low continuous	Moderate continuous	High continuous
**Overall population**						
Total # drinks trimester 1	0.040 ± 0.003	6.014 ± 0.109	19.683 ± 0.379	2.049 ± 0.151	25.130 ± 0.995	60.671 ± 4.505
Total # drinks trimester 2	0.018 ± 0.002	0.105 ± 0.130	0.300 ± 0.047	3.530 ± 0.130	7.501 ± 0.359	35.239 ± 2.921
Total # drinks trimester 3	0.011 ± 0.001	0.297 ± 0.004	0.146 ± 0.028	0.872 ± 0.054	2.918 ± 0.163	17.257 ± 1.776
Total # drinks in pregnancy	0.068 ± 0.004	6.148 ± 0.113	20.089 ± 0.386	6.448 ± 0.212	35.558 ± 0.851	113.167 ± 5.891
Total # binge events trimester 1	0.00 ± 0.00	0.43 ± 0.01	2.16 ± 0.03	0.11 ± 0.01	2.18 ± 0.10	5.71 ± 0.37
Total # binge events trimester 2	0.00 ± 0.00	0.00 ± 0.00	0.00 ± 0.00	0.26 ± 0.02	0.66 ± 0.04	3.79 ± 0.32
Total # binge events trimester 3	0.00 ± 0.00	0.00 ± 0.00	0.00 ± 0.00	0.00 ± 0.00	0.23 ± 0.02	1.67 ± 0.17
Total # binge events in pregnancy	0.00 ± 0.00	0.43 ± 0.01	2.16 ± 0.03	0.37 ± 0.03	3.07 ± 0.09	11.17 ± 0.53
**South Africa**						
Total # drinks trimester 1	0.032 ± 0.003	6.056 ± 0.156	18.856 ± 0.533	1.992 ± 0.156	19.996 ± 1.037	50.951 ± 3.889
Total # drinks trimester 2	0.024 ± 0.002	0.186 ± 0.026	0.476 ± 0.094	3.670 ± 0.139	9.081 ± 0.407	38.02 ± 3.118
Total # drinks trimester 3	0.013 ± 0.002	0.037 ± 0.008	0.257 ± 0.055	0.825 ± 0.056	3.463 ± 0.190	18.629 ± 1.913
Total # drinks in pregnancy	0.068 ± 0.004	6.280 ± 0.165	19.589 ± 0.552	6.517 ± 0.222	32.541 ± 0.925	107.600 ± 5.933
Total # binge events trimester 1	0.00 ± 0.00	0.47 ± 0.02	2.17 ± 0.04	0.11 ± 0.01	1.87 ± 0.10	5.21 ± 0.37
Total # binge events trimester 2	0.00 ± 0.00	0.00 ± 0.00	0.00 ± 0.00	0.27 ± 0.02	0.80 ± 0.04	4.11 ± 0.34
Total # binge events trimester 3	0.00 ± 0.00	0.00 ± 0.00	0.00 ± 0.00	0.00 ± 0.00	0.28 ± 0.02	1.79 ± 0.17
Total # binge events in pregnancy	0.00 ± 0.00	0.47 ± 0.01	2.17 ± 0.03	0.39 ± 0.03	2.59 ± 0.10	11.12 ± 0.57
**Northern Plains**						
Total # drinks trimester 1	0.052 ± 0.005	5.978 ± 0.152	20.340 ± 0.531	2.734 ± 0.613	45.667 ± 1.756	166.817 ± 24.181
Total # drinks trimester 2	0.008 ± 0.002	0.036 ± 0.007	0.743 ± 0.026	1.453 ± 0.330	1.220 ± 0.393	4.857 ± 3.628
Total # drinks trimester 3	0.007 ± 0.002	0.023 ± 0.005	0.049 ± 0.020	1.431 ± 0.188	0.737 ± 0.182	2.273 ± 1.397
Total # drinks in pregnancy	0.068 ± 0.004	6.280 ± 0.165	19.589 ± 0.552	6.517 ± 0.222	32.541 ± 0.925	173.947 ± 23.854
Total # binge events trimester 1	0.00 ± 0.00	0.39 ± 0.02	2.15 ± 0.04	0.07 ± 0.04	3.43 ± 0.20	11.12 ± 1.24
Total # binge events trimester 2	0.00 ± 0.00	0.00 ± 0.00	0.00 ± 0.00	0.07 ± 0.04	0.03 ± 0.02	0.32 ± 0.25
Total # binge events trimester 3	0.00 ± 0.00	0.00 ± 0.00	0.00 ± 0.00	0.00 ± 0.00	0.28 ± 0.02	1.79 ± 0.17
Total # binge events in pregnancy	0.00 ± 0.00	0.39 ± 0.01	2.10 ± 0.44	0.14 ± 0.06	3.57 ± 0.20	11.76 ± 1.18

**TABLE 4:** Number of cigarettes per week by trimester per smoking group in the overall population, South Africa population, and Northern Plains population.

	Smoking exposure			
	
	None	Quit early	Low continuous	Moderate/high continuous
**Overall population**				
Average # cigarettes/week in trimester 1	0.011 ± 0.0012	8.38 ± 0.57	15.57 ± 0.24	47.73 ± 0.70
Average # cigarettes/week in trimester 2	0.0025 ± 0.0005	0.74 ± 0.13	15.77 ± 0.27	50.48 ± 0.81
Average # cigarettes/week in trimester 3	0.0077 ± 0.0010	0.11 ± 0.014	14.75 ± 0.26	46.94 ± 0.80
Average # cigarettes/week in pregnancy	0.0071 ± 0.0006	2.86 ± 0.19	15.36 ± 0.22	48.38 ± 0.67
**South Africa**				
Average # cigarettes/week in trimester 1	0.008 ± 0.002	8.715 ± 0.574	16.003 ± 0.256	46.229 ± 0.732
Average # cigarettes/week in trimester 2	0.002 ± 0.001	0.100 ± 0.232	17.007 ± 0.283	50.156 ± 0.861
Average # cigarettes/week in trimester 3	0.006 ± 0.002	0.098 ± 0.021	15.999 ± 0.275	46.739 ± 0.828
Average # cigarettes/week in pregnancy	0.005 ± 0.001	2.971 ± 0.252	16.337 ± 0.229	47.708 ± 0.708
**Northern Plains**				
Average # cigarettes/week in trimester 1	0.0142 ± 0.002	8.111 ± 0.829	13.079 ± 0.678	59.759 ± 2.036
Average # cigarettes/week in trimester 2	0.003 ± 0.001	0.053 ± 0.014	8.596 ± 0.678	53.044 ± 2.524
Average # cigarettes/week in trimester 3	0.009 ± 0.001	0.115 ± 0.021	7.491 ± 0.566	48.5883 ± 2.751
Average # cigarettes/week in pregnancy	0.009 ± 0.001	2.760 ± 0.276	9.722 ± 0.511	53.797 ± 2.041

**TABLE 5:** Linear regression results from alcohol and smoking exposure predicting Mean HR in 1F.

Exposure category	Both sites			South Africa			Northern Plains		
			
	N	Effect size Mean difference (SE)	*p* val	N	Effect size Mean difference (SE)	*p* val	N	Effect size Mean difference (SE)	*p* val
No alcohol	1460	/	/	1027	/	/	433	/	/
Alcohol low quit early	682	−0.322 (0.375)	0.390	413	0.0197 (0.4607)	0.966	269	−0.9368 (0.6607)	0.157
Alcohol high quit early	146	1.135 (0.698)	0.104	84	2.170 (0.8989)	***0.016***	62	−0.346 (1.1396)	0.761
Alcohol low continuous	298	−0.217 (0.518)	0.675	284	0.0924 (0.5333)	0.863	14	−0.8672 (2.2564)	0.701
Alcohol moderate continuous	286	0.202 (0.536)	0.706	255	0.5537 (0.5722)	0.333	31	−0.8683 (1.5548)	0.577
Alcohol high continuous	135	1.431 (0.742)	0.054	129	2.0443 (0.7601)	***0.007***	6	−4.549 (3.4929)	0.193
No smoking	1641	/	/	950	/	/	691	/	/
Smoking quit early	96	1.909 (0.845)	***0.024***	53	0.9037 (1.115)	0.418	43	3.3568 (1.3482)	***0.013***
Smoking low continuous	766	−0.413 (0.390)	0.290	722	−0.6005 (0.4068)	0.140	44	−0.103 (1.3928)	0.941
Smoking moderate/high continuous	504	−0.682 (0.450)	0.130	467	−1.0197 (0.4743)	***0.0317***	37	1.0713 (1.4572)	0.4624

*SE: standard error; *P* val: *p*-values; *N* = number of participants in the group. Bold and italicized values represent p-value ≤ 0.05.*

**TABLE 6:** Linear regression results from alcohol and smoking exposure predicting Mean HR in 2F.

Exposure category	Both sites			South Africa			Northern Plains		
			
	N	Effect size Mean difference (SE)	*p* val	N	Effect size Mean difference (SE)	*p* val	N	Effect size Mean difference (SE)	*p* value
No alcohol	2774	/	/	1767	/	/	1007	/	/
Alcohol low quit early	1362	−0.043 (0.254)	0.867	682	0.367 (0.342)	0.283	680	−0.5566 (0.3832)	0.147
Alcohol high quit early	291	0.128 (0.467)	0.783	150	1.1246 (0.643)	0.0804	141	−0.9834 (0.6819)	0.149
Alcohol low continuous	530	−0.135 (0.367)	0.714	496	0.1759 (0.387)	0.6498	34	−0.8102 (1.32)	0.539
Alcohol moderate continuous	553	0.067 (0.370)	0.857	457	0.4089 (0.4085)	0.3169	76	−0.6608 (0.9086)	0.467
Alcohol high continuous	275	0.580 (0.492)	0.239	257	1.089 (0.518)	***0.0356***	18	−2.147 (1.803)	0.234
No smoking	3300	/	/	1634	/	/	1666	/	/
Smoking quit early	188	0.395 (0.569)	0.488	100	−0.433 (0.779)	0.578	88	1.271 (0.849)	0.135
Smoking low continuous	1394	−0.844 (0.271)	***0.0018***	1257	−1.0343 (0.295)	***0.0005***	137	−0.38 (0.7184)	0.597
Smoking moderate/high continuous	883	−1.252 (0.318)	***0.0001***	818	−1.510 (0.341)	***0.00001***	65	−0.0829 (0.9792)	0.933

*SE: standard error; *P* val: *p*-values; *N* = number of participants in the group. Bold and italicized values represent p-value ≤ 0.05.*

**TABLE 7:** Linear regression results from alcohol and smoking exposure predicting HR-SD in 1F.

Exposure category	Both sites			South Africa			Northern Plains		
			
	N	Effect size Mean difference (SE)	*p* val	N	Effect size Mean difference (SE)	*p* val	N	Effect size Mean difference (SE)	*p* val
No alcohol	1460	/	/	1027	/	/	433	/	/
Alcohol low quit early	682	0.0203 (0.034)	0.550	413	0.0413 (0.0421)	0.327	269	−0.0256 (0.0585)	0.661
Alcohol high quit early	146	0.0827 (0.0632)	0.191	84	0.1158 (0.0822)	0.159	62	0.0233 (0.1008)	0.818
Alcohol low continuous	298	0.0711 (0.0469)	0.130	284	0.0903 (0.0488)	0.0641	14	0.0125 (0.1997)	0.950
Alcohol moderate continuous	286	0.0973 (0.0486)	***0.045***	255	0.1336 (0.0523)	***0.0107***	31	−0.084 (0.1376)	0.542
Alcohol high continuous	135	−0.0706 (0.0672)	0.294	129	−0.0392 (0.0695)	0.5725	6	−0.2214 (0.3091)	0.474
No smoking	1641	/	/	950	/	/	691	/	/
Smoking quit early	96	0.0022 (0.0765)	0.977	53	−0.0655 (0.102)	0.521	43	0.0865 (0.1193)	0.469
Smoking low continuous	766	0.0197 (0.0353)	0.577	722	0.0004 (0.0372)	0.991	44	0.0653 (0.1232)	0.596
Smoking moderate/high continuous	504	−0.0236 (0.0407)	0.563	467	−0.0673 (0.0434)	0.121	37	0.2735 (0.1289)	***0.034***

*SE: standard error; *P* val: *p*-values; *N* = number of participants in the group. Bold and italicized values represent p-value ≤ 0.05.*

**TABLE 8:** Linear regression results from alcohol and smoking exposure predicting HR−SD in 2F.

Exposure category	Both sites			South Africa			Northern Plains		
			
	N	Effect size Mean difference (SE)	*p* val	N	Effect size Mean difference (SE)	*p* val	N	Effect size Mean difference (SE)	*p* val
No alcohol	2774	/	/	1767	/	/	1007	/	/
Alcohol low quit early	1362	0.00545 (0.0442)	0.902	682	−0.0001 (0.0555)	0.998	680	0.0407 (0.0740)	0.582
Alcohol high quit early	291	0.06202 (0.08102)	0.444	150	−0.0939 (0.1045)	0.369	141	0.2575 (0.1317)	0.051
Alcohol low continuous	530	0.00782 (0.0636)	0.902	496	−0.0324 (0.0629)	0.607	34	0.4756 (0.255)	0.062
Alcohol moderate continuous	553	−0.0146 (0.0642)	0.820	457	−0.0486 (0.0664)	0.464	76	0.1333 (0.1755)	0.448
Alcohol high continuous	275	0.0195 (0.0855)	0.820	257	0.0024 (0.0842)	0.977	18	0.0236 (0.3483)	0.946
No smoking	3300	/	/	1634	/	/	1666	/	/
Smoking quit early	188	−0.00164 (0.0988)	0.987	100	−0.0544 (0.126)	0.667	88	0.0171 (0.1641)	0.917
Smoking low continuous	1394	0.0216 (0.0470)	0.646	1257	0.0147 (0.0479)	0.759	137	0.1492 (0.1388)	0.283
Smoking moderate/high continuous	883	−0.0868 (0.0553)	0.116	818	−0.04703 (0.0555)	0.397	65	−0.3987 (0.1892)	***0.035***

*SE: standard error; *P* val: *p*-values; *N* = number of participants in the group. Bold and italicized values represent p-value ≤ 0.05.*

**TABLE 9:** Linear regression results from alcohol and smoking exposure predicting mean fetal movement in 1F.

Exposure category	Both sites			South Africa			Northern Plains		
			
	N	Effect size Mean difference (SE)	*p* val	N	Effect size Mean difference (SE)	*p* val	N	Effect size Mean difference (SE)	*p* val
No alcohol	1460	/	/	1027	/	/	433	/	/
Alcohol low quit early	682	−0.0384 (0.0524)	0.464	413	0.0116 (0.0673)	0.864	269	−0.1247 (0.0833)	0.135
Alcohol high quit early	146	0.1518 (0.0981)	0.122	84	0.135 (0.1319)	0.306	62	0.239 (0.1912)	0.212
Alcohol low continuous	298	0.0442 (0.0722)	0.541	284	0.0756 (0.0780)	0.332	14	−0.3511 (0.2723)	0.198
Alcohol moderate continuous	286	0.0230 (0.0748)	0.758	255	0.0291 (0.0836)	0.728	31	0.0899 (0.2796)	0.748
Alcohol high continuous	135	0.0847 (0.1036)	0.413	129	0.0865 (0.1113)	0.437	6	0.6305 (0.7516)	0.402
No smoking	1641	/	/	950	/	/	691	/	/
Smoking quit early	96	0.0822 (0.1175)	0.485	53	0.131 (0.1627)	0.421	43	0.003 (0.163)	0.985
Smoking low continuous	766	−0.0501 (0.0544)	0.358	722	−0.0391 (0.0594)	0.511	44	−0.0847 (0.1726)	0.624
Smoking moderate/high continuous	504	−0.1358 (0.0627)	***0.031***	467	−0.1085 (0.0694)	0.118	37	−0.3421 (0.1761)	0.052

*SE: standard error; *P* val: *p*-values; *N* = number of participants in the group. Bold and italicized values represent p-value ≤ 0.05.*

**TABLE 10:** Linear regression results from alcohol and smoking exposure predicting mean fetal movement in 2F.

Exposure category	Both sites			South Africa			Northern Plains		
			
	N	Effect size Mean difference (SE)	*p* val	N	Effect size Mean difference (SE)	*p* val	N	Effect size Mean difference (SE)	*p* val
No alcohol	2774	/	/	1767	/	/	1007	/	/
Alcohol low quit early	1362	−0.0861 (0.0392)	***0.028***	682	−0.0176 (0.0527)	0.738	680	−0.172 (0.06)	***0.004***
Alcohol high quit early	291	0.0486 (0.0723)	0.502	150	0.1008 (0.0993)	0.310	141	−0.049 (0.1329)	0.713
Alcohol low continuous	530	−0.0641 (0.0562)	0.254	496	−0.0341 (0.0597)	0.568	34	−0.1731 (0.2017)	0.391
Alcohol moderate continuous	553	0.0318 (0.0566)	0.574	457	0.0596 (0.063)	0.344	76	−0.0904 (0.1901)	0.635
Alcohol high continuous	275	0.0112 (0.0754)	0.137	257	0.1433 (0.0799)	0.073	18	−0.1994 (0.4537)	0.66
No smoking	3300	/	/	1634	/	/	1666	/	/
Smoking quit early	188	0.1095 (0.087)	0.208	100	0.2179 (0.1199)	0.069	88	−0.0455 (0.128)	0.722
Smoking low continuous	1394	−0.0258 (0.0415)	0.535	1257	−0.0172 (0.0455)	0.705	137	−0.0624 (0.1102)	0.571
Smoking moderate/high continuous	883	−0.1258 (0.0487)	***0.01***	818	−0.1047 (0.0526)	***0.047***	65	−0.2535 (0.1476)	0.086

*SE: standard error; *P* val: *p*-values; *N* = number of participants in the group. Bold and italicized values represent p-value ≤ 0.05.*

**TABLE 11:** Linear regression results from alcohol and smoking exposure predicting fetal movement/HR cross-correlation Lag.

Exposure category	Both sites			South Africa			Northern Plains		
			
	N	Effect size Mean difference (SE)	*p* val	N	Effect size Mean difference (SE)	*p* val	N	Effect size Mean difference (SE)	*p* val
No alcohol	2812	/	/	1709	/	/	1103	/	/
Alcohol low quit early	1473	−0.0399 (0.0793)	0.615	677	−0.129 (0.114)	0.257	760	0.0549 (0.1078)	0.611
Alcohol high quit early	310	0.0081 (0.144)	0.955	147	−0.331 (0.215)	0.124	163	0.3180 (0.1884)	0.092
Alcohol low continuous	506	−0.2655 (0.1188)	***0.025***	472	−0.288 (0.131)	***0.028***	34	−0.5247 (0.3903)	0.179
Alcohol moderate continuous	538	0.142 (0.116)	0.222	428	0.122 (0.139)	0.378	110	0.038 (0.226)	0.867
Alcohol high continuous	270	−0.0959 (0.1575)	0.543	248	−0.146 (0.174)	0.400	22	−0.2326 (0.4862)	0.632
No smoking	3372	/	/	1587	/	/	1785	/	/
Smoking quit early	222	0.1859 (0.1675)	0.267	105	0.0722 (0.252)	0.774	117	0.3031 (0.2203)	0.169
Smoking low continuous	1406	−0.0445 (0.0848)	0.600	1210	−0.0015 (0.0989)	0.987	196	−0.1401 (0.181)	0.439
Smoking moderate/high continuous	873	0.0497 (0.1005)	0.621	779	0.129 (0.115)	0.260	94	−0.3993 (0.2439)	0.102

*SE: standard error; *P* val: *p*-values; *N* = number of participants in the group. Bold and italicized values represent p-value ≤ 0.05.*

**TABLE 12:** Linear regression results from alcohol and smoking exposure predicting HR/fetal movement cross-correlation.

Exposure category	Both sites			South Africa			Northern Plains		
			
	N	Effect size Mean difference (SE)	*p* val	N	Effect size Mean difference (SE)	*p* val	N	Effect size Mean difference (SE)	*p* val
No alcohol	2812	/	/	1709	/	/	1103	/	/
Alcohol low quit early	1473	0.0031 (0.0035)	0.375	677	0.0035 (0.0048)	0.471	760	0.0028 (0.0052)	0.590
Alcohol high quit early	310	−0.0022 (0.0064)	0.727	147	−0.0171 (0.0091)	0.061	163	0.0109 (0.0091)	0.229
Alcohol low continuous	506	0.0081 (0.0053)	0.127	472	0.0064 (0.0056)	0.255	34	0.0098 (0.0188)	0.604
Alcohol moderate continuous	538	0.000061 (0.0052)	0.991	428	−0.0042 (0.0059)	0.481	110	0.0122 (0.0109)	0.261
Alcohol high continuous	270	−0.0032 (0.00701)	0.647	248	−0.0028 (0.0074)	0.704	22	−0.0269 (0.0234)	0.251
No smoking	3372	/	/	1587	/	/	1785	/	/
Smoking quit early	222	−0.0109 (0.0075)	0.142	105	−0.0148 (0.0107)	0.166	117	−0.0049 (0.0106)	0.646
Smoking low continuous	1406	−0.00205 (0.00377)	0.587	1210	0.00095 (0.0042)	0.821	196	−0.0132 (0.0087	0.132
Smoking moderate/high continuous	873	0.00203 (0.00447)	0.650	779	0.0026 (0.0049)	0.593	94	0.0071 (0.0118)	0.547

### Significant Associations of Covariates With Fetal Physiology

In the analysis with *sites combined*, sex, GA at assessment and site were significantly associated with mean HR in 1F (Higher HR in females, β = 0.85 ± 0.29, *p* = 0.0036; decreasing HR with increasing GA, β = −0.10 ± 0.02, *p* < 0.001; lower HR in the Northern Plains β = −2.23 ± 0.55, *p* < 0.001), and with mean HR in state 2F (Higher HR in females, β = 1.06 ± 0.20, *p* < 0.001; decreasing HR with increasing GA β = −0.025 ± 0.012, *p* = 0.041; lower HR in the Northern Plains β = −2.63 ± 0.37, *p* < 0.001).

Sex, GA at assessment and site were all significantly related to HR-SD in both fetal states. In state 1F males had higher HR-SD than females (β = −0.06 ± 0.026, *p* = 0.023), HR-SD decreased with age (β = −0.07 ± 0.002, *p* < 0.001), and HR-SD was higher in fetuses from the Northern Plains (β = 0.18 ± 0.05; *p* < 0.001). Each of these findings were also seen in state 2F (β = −0.10 ± 0.03, *p* = 0.003; β = 0.01 ± 0.002, *p* < 0.001; β = 0.43 ± 0.06, *p* < 0.001, respectively).

Site was significantly associated with fetal movement in state 1F with mean levels of movement greater in the South Africa cohort (β = −0.19 ± 0.08, *p* = 0.017).

For fetal HR/movement cross-correlation, GA at assessment and site were significant predictor (higher values with increasing GA β = 0.0008 ± 0.0002, *p* < 0.001; Lower values in the Northern Plains β = −0.017 ± 0.0047, *p* < 0.001), whereas for the lag of the cross-correlation sex and CI were significant (lower values for females, β = −0.18 ± 0.06, *p* = 0.0036; β = −0.86 ± 0.41, *p* = 0.034; β = −1.00 ± 0.40, *p* = 0.013; β = −0.94 ± 0.41, *p* = 0.020).

In *South Africa,* sex, GA at assessment and depression were significant predictors of mean HR in 1F (Higher HR for females, β = 0.73 ± 0.34, *p* = 0.03; decreasing HR with increasing GA, β = −0.10 ± 0.02, *p* < 0.001; decreasing HR with increasing depression, β = −0.06 ± 0.03, *p* = 0.025). Sex, depression and CI 25–75th were significant predictors of mean HR in 2F (respectively, β = 0.89 ± 0.24, *p* < 0.001; β = −0.04 ± 0.02, *p* < 0.04; β = 3.42 ± 1.74, *p* < 0.049). Sex was also a significant predictor of HR SD in 1F (Lower HR SD for females, β = −0.06 ± 0.03, *p* = 0.037), while sex and GA at assessment were significant for HR SD in 2F (Lower HR SD for females, β = −0.088 ± 0.040, *p* = 0.027, increasing HR SD with increasing GA β = 0.01 ± 0.003, *p* < 0.001). No additional significant predictors were found for mean fetal movement. For fetal HR/movement cross-correlation, GA at assessment was significant (increasing values with increasing GA, β = 0.0006 ± 0.0002, *p* < 0.0121), whereas for the lag of the cross-correlation sex and CI were significant (lower values for females, β = −0.20 ± 0.08, *p* = 0.01; β = −1.27 ± 0.58, *p* = 0.027; β = −1.24 ± 0.55, *p* = 0.023; β = −1.19 ± 0.55, *p* = 0.030).

In the *Northern Plains,* sex and GA at assessment were significant predictors of mean HR in 1F (respectively, β = 1.37 ± 0.58, *p* = 0.019; β = −0.10 ± 0.03, *p* < 0.0016), and sex and CI were significant predictors of mean HR in 2F (β = 1.42 ± 0.34, *p* < 0.001, β = 3.91 ± 1.96, *p* = 0.047; β = 3.94 ± 1.98, *p* = 0.047). GA at assessment was a significant predictor of HR std in 1F (β = −0.01 ± 0.003, *p* < 0.001), and sex, GA at assessment and education level 1 and 2 were significant predictors for HR-SD in 2F (β = −0.13 ± 0.07, *p* = 0.041; β = 0.01 ± 0.003, *p* = 0.0021; β = 0.78 ± 0.35, *p* = 0.025; β = 0.29 ± 0.13, *p* = 0.023). Sex was a significant predictor of fetal movement in 1F (β = 0.16 ± 0.07, *p* = 0.023) and GA at assessment in 2F (β = −0.009 ± 0.003, *p* < 0.001). Similarly, GA at assessment was significant for fetal HR/movement cross-correlations (β = 0.001 ± 0.0002, *p* < 0.001).

In summary, expected findings of sex on autonomic regulation were observed, with females having higher HR and lower HR-SD. GA at assessment was also significant in many associations that are consistent with the literature, with HR decreasing and variability increasing with GA (Burtchen et al., 2019). Depression was also an important covariate, with increasing values of the EDPS associated with reduced HR in SA, where many mothers presented with high scores on the EPDS questionnaire. Lastly, we observed that site was significantly associated with autonomic measures, with fetuses in SA having higher HR and lower variability compared to the NP.

### Effects of Prenatal Tobacco Exposure

**FIGURE 1:**
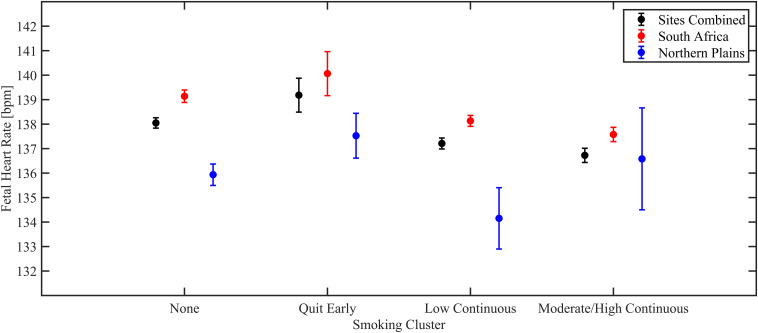
Estimated marginal means from linear regression models of mean fetal HR in 2F, shown for the overall population (black), South Africa (red), and Northern Plains (blue).

In the analyses considering both *sites combined*, we found a significant association between smoking and mean HR in state 2F. A dose response effect was observed, with the low continuous group having a decrease of 0.84 ± 0.27 beats per minute (BPM, Mean ± SD) compared to non-smokers (*p* = 0.0018) and the moderate/high continuous group having a decrease of 1.25 ± 0.32 BPM (*p* = 0.0001) compared to non-smokers. Women who quit in the first trimester were not significantly different from non-smokers. These results are shown in Figure 1. We also found a significant association between smoking and mean HR in state 1F, but only for women who quit before the end of the first trimester. The HR of their fetuses was 1.91 ± 0.9 BPM per minute higher compared to the unexposed group (*p* = 0.024).

**FIGURE 2:**
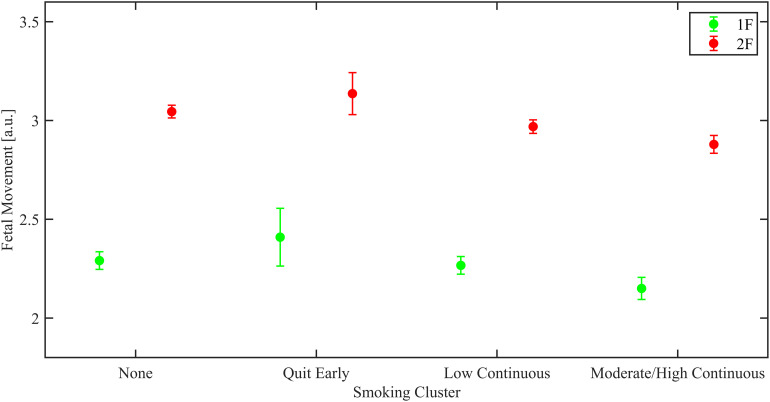
Estimated marginal means from linear regression models of mean fetal movement, shown for the overall population by fetal behavioral sleep state (green 1F and red 2F).

Smoking was also significantly associated with fetal movement in both states 1F and 2F. The moderate/high continuous group showed lower fetal movement compared to the non-smokers (1F: decrease of 0.14 ± 0.06 a.u., *p* = 0.031; 2F decrease of 0.13 ± 0.05 a.u., *p* = 0.01). These results are portrayed in Figure 2.

In *South Africa*, similar significant associations with smoking were observed for mean HR in state 2F, with a dose response reduction observed (Low continuous group decrease of 1.03 ± 0.30 BPM, *p* < 0.001; Moderate/high continuous group decrease of 1.51 ± 0.34 BPM, *p* < 0.001). A significant association with smoking was also observed in state 1F, with a decrease of 1.02 ± 0.47 BPM in mean HR for the moderate/high continuous group (*p* = 0.032).

Regarding the association of smoking and fetal movement, in South Africa subjects there was as significant reduction in fetal movement in the moderate/high group only in 2F was observed (decrease of 0.10 ± 0.05 a.u., *p* = 0.047).

In the *Northern Plains*, there was a significant increase in mean HR of 3.36 ± 1.35 BPM in 1F in fetuses of women who quit smoking in the first trimester (*p* = 0.013). In addition, there was a significant increase in HR-SD in 1F in the smoking moderate/high continuous group compared to the non-smokers (increase of 0.27 ± 0.13 BPM, *p* = 0.034), while a significant decrease in HR-SD was observed in 2F for smoking moderate/high continuous group (decrease of 0.40 ± 0.19 BPM, *p* = 0.035). In site-combined analyses similar trends to the combined were observed. Specifically, there was a reduction of fetal movement for the moderate/high group in 1F, but it did not reach significance (decrease of 0.34 ± 0.18 a.u., *p* = 0.052).

### Effects of Prenatal Alcohol Exposure

In the dataset with both sites combined, we found a significant association between PAE and the HR-SD in 1F, with an increase for the moderate continuous group by 0.10 ± 0.05 BPM (*p* = 0.045). Additionally, we found a significant reduction in fetal movement in 2F for the low quit early group (decrease of 0.09 ± 0.04 a.u., *p* = 0.028) compared to the non-drinkers.

The fetal movement/HR cross-correlation lag of the low continuous group was 0.27 s shorter than the non-drinkers (β = −0.27 ± 0.12, *p* = 0.025).

**FIGURE 3:**
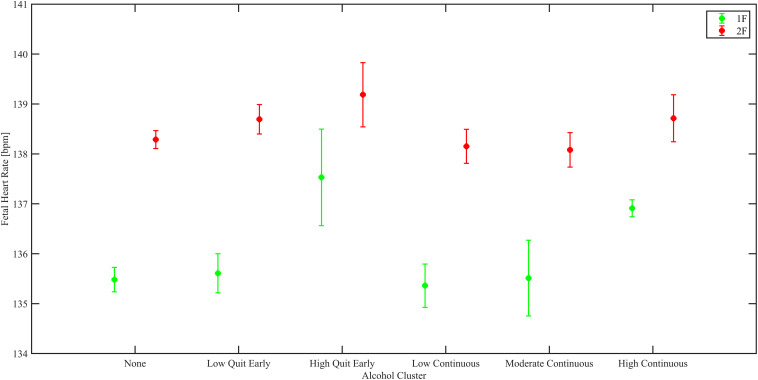
Estimated marginal means from linear regression models of mean fetal HR, shown for the SA population by fetal behavioral sleep state (green 1F and red 2F).

In the *South Africa* dataset, we found an increase in mean HR in 1F both for the high quit early and the high continuous group (respectively, increase of 2.17 ± 0.90 BPM, *p* = 0.016; 2.04 ± 0.76 BPM, *p* = 0.007). Similarly, in 2F the mean HR of the high continuous group was elevated compared to the non-drinkers (increase of 1.09 ± 0.52 BPM, *p* = 0.036). Results are shown in Figure 3. The moderate continuous alcohol group had a higher HR-SD in 1F (increase of 0.13 ± 0.05 BPM, *p* = 0.011) compared to non-drinkers.

The low continuous group showed shorter cross-correlation lag times between movement and change in HR than the non-drinkers (β = −0.29 ± 0.13, *p* = 0.028).

In the *Northern Plains*, we only observed a significant a decrease in fetal movement in 2F in the low quit early group compared to non-drinkers (decrease of 0.17 ± 0.06 a.u., *p* = 0.004).

## Discussion

Several studies have reported associations of smoking and drinking during pregnancy with negative gestational outcomes and health in offspring (Schoendorf and Kiely, 1992; Scragg et al., 1993; Cnattingius, 2004; Mamluk et al., 2017; Shuffrey et al., 2020). Although these reports clearly demonstrate there are adverse effects of PAE and PTE, they do not provide information about the contributions of timing and amount of exposure on fetal ANS function. This current study addresses that shortcoming by focusing on assessments of fetal HR, HR-SD, and movement during weeks 34 to 38 of pregnancy. To our knowledge, the Safe Passage Study data set is unique in size, details of exposures, and breadth of subject characteristics.

### Prenatal Tobacco Exposure

In this study, PTE was associated with a decrease in mean HR in fetal state 2F, both in the overall population and in the sub-analysis on the SA population. These effects appeared to be dependent on dose, in that the mean decreases in HR were greatest in the fetuses of mothers who smoked at the highest levels and were not significant in fetuses of women who quit smoking during the first trimester. There were no significant PTE exposure effects in state 2F in the Northern Plans cohort; however, the number of subjects in the highest exposure group in the NP was only 65 as compared to 818 in SA. In state 1F, overall, there a significant *increase* in fetal HR in fetuses whose mothers smoked but quit during the first trimester. Broken down by site, this effect was significant only in the NP cohort. A decrease in HR in state 2F with high exposure but only in SA, and an increase in HR in state 1F in subjects whose mothers quit smoking early in pregnancy, but only in the NP suggests there are site specific factors that interact with PTE exposure, though our analyses did not reveal what these might be.

Combining both sites there were no significant effects of PTE on HR-SD. However, in the NP, the moderate-high continuous groups showed an increase in HR-SD in 1F and a decrease in 2F. The fact that PTE was associated with divergent effects in the two fetal sleep states and only in the NP site was unexpected. However, this effect might represent less differentiated state dependent autonomic activity in some populations. In both sleep states in the combined data set PTE was also associated with a significant reduction in fetal movement in the most highly exposed group. This association was significant or approaching significance at both sites.

Tobacco cigarette smoke contains several substances which can potentially be harmful to the fetus, of these, nicotine is the most studied. Nicotine enters in the mother’s bloodstream quickly and easily crosses the placenta into the fetal bloodstream (Slotkin, 1998; Cohen et al., 2005). High levels of nicotine on the fetal side of the placenta can result in a variety of adverse effects on the developing fetus (Duncan et al., 2009). Prenatal exposure to nicotine may induce functional alterations in neuronal differentiation including changes in hippocampal, cerebellar, and sensory cortex development (Shuffrey and Fifer, 2020). Previous studies looking at the acute effects of smoking on fetal autonomic regulation reported mixed findings (Lehtovirta et al., 1983; Goodman et al., 1984; Kelly et al., 1984; Ates et al., 2004; Cowperthwaite et al., 2007). Part of the reasons for the disparate findings can be attributed to the small sample size and the inconsistent characterization of patterns of smoking. Additionally, some studies investigated the acute effect of smoking while very few addressed the effect of chronic exposure. When acute effects of smoking were investigated most studies found an increase in fetal HR paired with a decrease in fetal HRV and reactivity (Goodman et al., 1984; Oncken et al., 2002), suggesting that smoking causes increased sympathetic activity.

Results from our study address the chronic effect of smoking exposure during pregnancy, which seems to go in the opposite direction of most acute studies, with a decrease in mean HR usually interpreted as a result of parasympathetic activation or sympathetic inhibition (or a combination of the two). There are few studies assessing fetuses chronically exposed to cigarette smoke. In one small study (*n* = 13 exposed, 13 controls), exposed fetuses were observed to spend more time in a low fetal HR variation pattern and fetal activity was decreased (Coppens et al., 2001), the latter finding in agreement with our own. Kapaya et al., also found results similar to those presented in this article, with a significantly lower HR baseline in fetuses of smokers (Kapaya et al., 2014). Analogously, Duncan et al. found that fetal baboons with chronic exposure to nicotine showed an increased parasympathetic control of the heart with an increase in high-frequency HRV. These changes in HRV were associated with abnormal 5-HT-nicotine alterations in the raphe’ obscurus and increased nicotinic receptor binding in the raphe’ obscurus and vagal complex in the nicotine-exposed animals (Duncan et al., 2009). Prenatal exposure to maternal smoking may also result in reduced fetal oxygenation. Pathology evaluations of the placentas of smokers have shown structural changes, including a reduction in the fraction of capillary volume and increased thickness of the villous membrane when compared with non-smokers (Burton et al., 1989; Jauniaux and Burton, 1992; Larsen et al., 2002). Both factors may contribute to abnormal gas exchange within the placenta and could explain the reduced fetal movement observed in our findings (Bocking, 2003). Furthermore, it is well-known that smoking can affect fetal growth, increasing the risk for fetal growth restriction (Reeves and Bernstein, 2008). In fetuses affected by fetal growth restriction a reduction in fetal movements has been observe, potentially to conserve energy (Baschat et al., 2001). A decline in fetal movements may lead to fewer accelerations, which could induce the observed reduction in the mean HR. It is noteworthy that mean changes in fetal HR and other variables associated with smoking were small and not of immediate clinically significance in and of themselves. However, these current results support the view that chronic prenatal smoking exposure shifts the cardiac autonomic regulation to favor inhibitory actions on cardiac function.

Importantly, from a public health perspective, HR parameters of fetuses whose mothers quit smoking by the end of first trimester were not significantly different from those of non-smokers. This is in line with epidemiological findings showing that risk of stillbirth to mothers who stopped smoking during the first trimester was comparable to the risk among women who were non-smokers during the entire pregnancy (Wisborg et al., 2001) and similarly mothers who quit smoking in the first trimester have a reduced risk of preterm delivery compared to those who continued to smoke (Mainous and Hueston, 1994). Additionally, quitting before 12 weeks GA was found to diminish differences in fetal growth in comparison to non-smokers (Vardavas et al., 2010). Thus, it appears that smoking is more harmful to the developing fetus during the latter part of gestation and these findings reinforce the importance of smoking cessation early during pregnancy.

### Prenatal Alcohol Exposure

In this study there were few significant findings regarding the associations of alcohol and fetal physiology. While differences in findings between sites for the high continuous group could be due to different distributions of participants across exposure groups, it is worth noting that similar results were not observed in the high quit early groups, which had similar number of subjects in the two sites. One possible interpretation for the different site findings, is differential rates of alcohol metabolization, potentially related to body mass index (BMI). Mothers’ diet can affect the fraction of body mass composed by adipose tissue, which is relevant since ingested alcohol distributes through the body water differently between lean and fat body mass (Reed, 1978).

To our knowledge, no previous studies have investigated the effects of chronic alcohol consumption during pregnancy on ANS function. Nonetheless, a few studies have investigated other aspects of fetal neurobehavior, such as behavioral states, and spontaneous and elicited startles. Hepper et al. showed that alcohol consumption delayed the decrease in the incidence of fetal startles observed with normal development. Regarding elicited startles, they found instead that fetuses exposed to alcohol were less likely to startle in response to sound than fetuses of non-drinkers (Hepper, 2007). Another relevant study from Haley et al. reported similar results to what we found but in 5–7 months old infants, who showed higher HR when exposed to high frequency drinking prenatally (Haley et al., 2006). Thus, these findings are convergent with results observed in the South Africa cohort were alterations in autonomic regulation were observed in fetuses exposed to high levels of alcohol, even if only in the first trimester.

PAE and PTE are risk factors for adverse fetal and neonatal outcomes such as intra uterine growth restriction, SIDS, and these same outcomes have been associated with altered ANS profiles (Pincus et al., 1993; Matturri and Lavezzi, 2011; Pini et al., 2021). Thus, the alterations of fetal physiology associated with PTE and PAE we describe could inform our understanding of the possible mechanisms linking PTE and PAE and adverse fetal and infant outcome. In addition, a large body of research has stressed the profound importance of the fetal environment in “programming” postnatal neurobehavioral and medical outcomes (Godfrey and Barker, 2001). The dominant theory suggests that fetuses adapt their physiology to cope with stressful environments and that, while effective in the short term these adaptations may predispose offspring to increased long term morbidity or mortality (Cao-Lei et al., 2017). Fetal HR and movement are measures of fetal well-being and maturation and have been found to be associated with later neurodevelopment (DiPietro et al., 2007; Voegtline et al., 2016). Thus, is it also possible that changes in these physiological systems in response to drinking and/or smoking are adaptations to these exposures and that adverse postnatal consequences reflect this adaptation. Regardless of mechanism, even small shifts in physiology move more individuals into low or high regions of the normal distribution. We speculate that these shifts to extreme values underly and/or are correlated with adverse outcomes associated with these toxic exposures.”

Limitations of this study include the possible under-reporting of PTE and PAE due to the use of self-report measure and the lack of information on acute smoking in recordings from mothers in the low, moderate and high continuous group. We do not know the precise interval between the last cigarette that the mothers smoked and the fetal assessment. Nonetheless, given the typical time required to transport the participant and prepare for the study protocol, it is highly unlikely that women smoked a cigarette in the hour before fetal monitoring. Another limitation is the lack of precise information on time of the day of assessments, which could affect HR since fetuses start to show circadian autonomic regulation during the third trimester. In addition, part of the fetal data collection occurred while mothers were responding to questionnaires, which could have affected maternal and fetal HR regulation. Lastly, our data could reflect a potential selection bias, since the effect of alcohol and smoking on fetal autonomic parameters were not investigated in adverse pregnancy outcomes such as early delivery or fetal demise.

In conclusion, this investigation addresses a significant gap in the literature on the association smoking and drinking during pregnancy with fetal autonomic regulation. To our knowledge, this study is unique both due to the size of the cohort and the comprehensive characterization of patterns of PTE and PAE, summarized in data driven exposure groups, taking into account both timing and magnitude of exposure. We believe these results can contribute to identifying biomarkers and potentially understanding the mechanisms underlying risk for adverse outcomes.

## Data Availability Statement

The raw data supporting the conclusions of this article will be made available by the authors, without undue reservation.

## Ethics Statement

The studies involving human participants were reviewed and approved by Health Research Ethics Committee of Stellenbosch University, Sanford Health’s Institutional Review Board, New York State Psychiatric Institute of Institutional Review Board, and Indian Health Service Institutional Review Board. Written informed consent to participate in this study was provided by the participants’ legal guardian/next of kin.

## Author Contributions

ML, LS, NP, AS, MM, WF, HO, and AE contributed to the conception and design of the study. LS, CP, CF, JA, LB, LTB, and CG contributed to the acquisition. ML, JN, LS, AS, NP, and MN contributed to the analysis of data. All authors significantly contributed to the interpretation of the data and drafting the article.

## Conflict of Interest

The authors declare that the research was conducted in the absence of any commercial or financial relationships that could be construed as a potential conflict of interest.

## Publisher’s Note

All claims expressed in this article are solely those of the authors and do not necessarily represent those of their affiliated organizations, or those of the publisher, the editors and the reviewers. Any product that may be evaluated in this article, or claim that may be made by its manufacturer, is not guaranteed or endorsed by the publisher.

## Supplementary Material

Supplementary Figure 1 Study flowchart.

Supplementary Material 1 Missing data imputation.

Supplementary Material 2 Alcohol and smoking cluster analysis.

Supplementary Material 3 Data processing.

Supplementary Material 4 Fetal state coding.

## References

[B1] Acharya U. R. Joseph K. P. Kannathal N. Lim C. M. Suri J. S. (2006). Heart rate variability: a review. *Med. Biol. Eng. Comput.* 44 1031–1051. 17111118 10.1007/s11517-006-0119-0

[B2] American College of Obstetricians and Gynecologitsts (2017). Committee opinion No. 721. smoking cessation during pregnancy. *Obstet. Gynecol.* 130 e200–e204. e200-4, 10.1097/AOG.0000000000002353 28937573

[B3] Ates U. Ata B. Armagan F. Has R. Sidal B. (2004). Acute effects of maternal smoking on fetal hemodynamics. *Int. J. Gynecol. Obstet.* 87 14–18. 10.1016/j.ijgo.2004.06.009 15464770

[B4] Bailey B. A. Sokol R. J. (2011). Prenatal alcohol exposure and miscarriage, stillbirth, preterm delivery, and sudden infant death syndrome. *Alcohol Res. Health* 34 86–91. 23580045 PMC3860553

[B5] Barrett J. M. Vanhooydonk J. E. Boehm F. H. (1981). Acute effect of cigarette smoking on the fetal heart nonstress test. *Obstet. Gynecol.* 57 422–425. 7243086

[B6] Baschat A. A. Gembruch U. Harman C. R. (2001). The sequence of changes in Doppler and biophysical parameters as severe fetal growth restriction worsens. *Ultrasound Obstet. Gynecol.* 18 571–577. 10.1046/j.0960-7692.2001.00591.x 11844191

[B7] Bocking A. D. (2003). Assessment of fetal heart rate and fetal movements in detecting oxygen deprivation in-utero. *Eur. J. Obstet. Gynecol. Reprod. Biol.* 110 S108–S112. 12965098 10.1016/s0301-2115(03)00180-5

[B8] Bulterys M. (1990). High incidence of sudden infant death syndrome among northern Indians and Alaska natives compared with southwestern Indians: possible role of smoking. *J. Commun. Health* 15 185–194. 10.1007/bf01350256 2365840

[B9] Burton G. J. Palmer M. E. Dalton K. J. (1989). Morphometric differences between the placental vasculature of non-smokers, smokers and ex-smokers. *Br. J. Obstet. Gynaecol.* 96 907–915. 10.1111/j.1471-0528.1989.tb03344.x 2775688

[B10] Burtchen N. Myers M. M. Lucchini M. Retamar M. O. Rodriguez D. Fifer W. P. (2019). Autonomic signatures of late preterm, early term, and full term neonates during early postnatal life. *Early Hum. Dev.* 137:104817. 10.1016/j.earlhumdev.2019.06.012 31352221

[B11] Cao-Lei L. De Rooij S. R. King S. Matthews S. G. Metz G. A. S. Roseboom T. J. (2017). Prenatal stress and epigenetics. *Neurosci. Biobehav. Rev.* 117 198–210. 28528960 10.1016/j.neubiorev.2017.05.016

[B12] Cnattingius S. (2004). The epidemiology of smoking during pregnancy?: smoking prevalence, maternal characteristics, and pregnancy outcomes. *Nicotine Tob. Res.* 6 S125–S140. 15203816 10.1080/14622200410001669187

[B13] Cohen G. Roux J.-C. Grailhe R. Malcolm G. Changeux J.-P. Lagercrantz H. (2005). Perinatal exposure to nicotine causes deficits associated with a loss of nicotinic receptor function. *Proc. Natl. Acad. Sci. U. S. A.* 102 3817–3821. 10.1073/pnas.0409782102 15738419 PMC552781

[B14] Coppens M. Vindla S. James D. K. Sahota D. S. (2001). Computerized analysis of acute and chronic changes in fetal heart rate variation and fetal activity in association with maternal smoking. *Am. J. Obstet. Gynecol.* 185 421–426. 10.1067/mob.2001.115992 11518903

[B15] Cowperthwaite B. Hains S. M. J. Kisilevsky B. S. (2007). Fetal behavior in smoking compared to non-smoking pregnant women. *Infant Behav. Dev.* 30 422–430. 10.1016/j.infbeh.2006.12.004 17683752

[B16] Cox J. L. Holden J. M. Sagovsky R. (1987). Detection of postnatal depression: development of the 10-item Edinburgh postnatal depression scale. *Br. J. Psychiatry* 150 782–786. 10.1192/bjp.150.6.782 3651732

[B17] DiPietro J. A. Bornstein M. H. Hahn C. S. Costigan K. Achy-Brou A. (2007). Fetal heart rate and variability: stability and prediction to developmental outcomes in early childhood. *Child Dev.* 78 1788–1798. 10.1111/j.1467-8624.2007.01099.x 17988321 PMC2267766

[B18] Dipietro J. A. Costigan K. A. Voegtline K. M. (2015). Studies in fetal behavior: revisited, renewed, and reimagined. *Monogr. Soc. Res. Child Dev.* 80 1–104. 10.1111/mono.v80.3 PMC4835043 26303396

[B19] Dipietro J. A. Irizarry R. A. Hawkins M. Costigan K. A. Pressman E. K. (2001). Cross-correlation of fetal cardiac and somatic activity as an indicator of antenatal neural development. *Am. J. Obstet. Gynecol.* 185 1421–1428. 10.1067/mob.2001.119108 11744919

[B20] Dukes K. Tripp T. Petersen J. Robinson F. Odendaal H. Elliott A. (2017). A modified Timeline followback assessment to capture alcohol exposure in pregnant women: application in the safe passage study. *Alcohol* 62(Suppl. C) 17–27. 10.1016/j.alcohol.2017.02.174 28755748 PMC5553051

[B21] Dukes K. A. Burd L. Elliott A. J. Fifer W. P. Folkerth R. D. Hankins G. D. V. V. (2014). The safe passage study: design, methods, recruitment, and follow-up approach. *Paediatr. Perinat. Epidemiol.* 28 455–465. 10.1111/ppe.12136 25131605 PMC4286367

[B22] Duncan J. R. Garland M. Myers M. M. Fifer W. P. Yang M. Kinney H. C. (2009). Prenatal nicotine-exposure alters fetal autonomic activity and medullary neurotransmitter receptors: implications for sudden infant death syndrome. *J. Appl. Physiol.* 107 1579–1590. 10.1152/japplphysiol.91629.2008 19729586 PMC2777800

[B23] Eriksen P. S. Gennser G. Lindvall R. Nilsson K. (1984). Acute effects of maternal smoking on fetal heart beat intervals. *Acta Obstet. Gynecol. Scand.* 63 385–390. 10.3109/00016348409156689 6496041

[B24] Gennser G. Maršál K. Brantmark B. (1975). Maternal smoking and fetal breathing movements. *Am. J. Obstet. Gynecol.* 123 861–867. 10.1016/0002-9378(75)90863-7 1106202

[B25] Godfrey K. M. Barker D. J. P. (2001). Fetal programming and adult health. *Public Health Nutr.* 4 611–624. 11683554 10.1079/phn2001145

[B26] Goodman J. D. S. Visser F. G. A. Dawes G. S. (1984). Effects of maternal cigarette smoking on fetal trunk move- ments, fetal breathing movements and the fetal heart rate. *Br. J. Obstet. Gynaecol.* 91 657–661. 10.1111/j.1471-0528.1984.tb04826.x 6743607

[B27] Haley D. W. Handmaker N. S. Lowe J. (2006). Infant stress reactivity and prenatal alcohol exposure. *Alcohol Clin. Exp. Res.* 30 2055–2064. 10.1111/j.1530-0277.2006.00251.x 17117971

[B28] Halmesmaki E. Ylikorkala O. (1986). The effect of maternal ethanol intoxication on fetal cardiotocography: a report of four cases. *Br. J. Obstet. Gynaecol.* 93 203–205. 10.1111/j.1471-0528.1986.tb07893.x 3964593

[B29] Hepper P. G. (2007). The effect of maternal consumption of alcohol on the behavior of the human fetus: a review. *Int. J. Disabil. Hum. Dev.* 6 153–160.

[B30] Himes S. K. Dukes K. A. Tripp T. Petersen J. M. Raffo C. Burd L. (2015). Clinical sensitivity and specificity of meconium fatty acid ethyl ester, ethyl glucuronide, and ethyl sulfate for detecting maternal drinking during pregnancy. *Clin. Chem.* 61 523–532. 10.1373/clinchem.2014.233718 25595440 PMC4485427

[B31] Huang L. Wang Y. Zhang L. Zheng Z. Zhu T. Qu Y. (2018). Maternal smoking and attention-deficit/hyperactivity disorder in offspring: a meta-analysis. *Pediatrics* 141:e20172465. 10.1542/peds.2017-2465 29288161

[B32] International Alliance for Responsible Drinking (Iard). (2019). *Drinking Guidelines for Pregnancy and Breastfeeding.* Available online at: http://iardwebprod.azurewebsites.net/science-resources/detail/Drinking-Guidelines-for-Pregnancy-and-Breastfeedin (accessed June, 2020).

[B33] Iyasu S. (2002). Risk factors for sudden infant death syndrome among northern plains Indians. *JAMA* 288 2717–2723. 10.1001/jama.288.21.2717 12460095

[B34] Jansson L. M. Dipietro J. Elko A. (2005). Fetal response to maternal methadone administration. *Am. J. Obstet. Gynecol.* 193 611–617. 10.1016/j.ajog.2005.02.075 16150250

[B35] Jauniaux E. Burton G. J. (1992). The effect of smoking in pregnancy on early placental morphology. *Obstet. Gynecol.* 79 645–648. 1565343

[B36] Kapaya H. Broughton-pipkin F. Hayes-gill B. Pamela V. (2014). Smoking in pregnancy affects the fetal heart?: possible links to future cardiovascular disease. *J. Matern. Neonatal Med.* 28 1664–1668. 10.3109/14767058.2014.964202 25212975

[B37] Kelly J. Mathews K. A. O’Conor M. (1984). Smoking in pregnancy?: effects on mother and fetus. *Br. J. Obstet. Gynaecol.* 91 111–117. 10.1111/j.1471-0528.1984.tb05892.x 6696855

[B38] Klonoff-Cohen H. Lam-Kruglick P. (2001). Maternal and paternal recreational drug use and sudden infant death syndrome. *Arch. Pediatr. Adolesc. Med.* 155 765–770. 10.1001/archpedi.155.7.765 11434841

[B39] Larsen L. G. Clausen H. V. Jønsson L. (2002). Stereologic examination of placentas from mothers who smoke during pregnancy. *Am. J. Obstet. Gynecol.* 186 531–537. 10.1067/mob.2002.120481 11904619

[B40] Lehtovirta P. Forss M. Rauramo I. Kariniemi V. (1983). Acute effects of nicotine on fetal heart rate variability. *Br. J. Obstet. Gynaecol.* 90 710–715. 6882704 10.1111/j.1471-0528.1983.tb09299.x

[B41] Mainous A. G. III Hueston W. J. (1994). The effect of smoking cessation during pregnancy on preterm delivery and low birthweight. *J. Fam. Pract.* 38 262–266. 8126407

[B42] Mamluk L. Edwards H. B. Savovi J. Leach V. Jones T. Moore T. H. M. (2017). Low alcohol consumption and pregnancy and childhood outcomes?: time to change guidelines indicating apparently ‘safe’ levels of alcohol during pregnancy? A systematic review and meta-analyses. *BMJ Open.* 7:e015410. 10.1136/bmjopen-2016-015410 28775124 PMC5642770

[B43] Matturri L. Lavezzi A. M. (2011). Unexplained stillbirth versus SIDS: common congenital diseases of the autonomic nervous system—pathology and nosology. *Early Hum. Dev.* 87 209–215. 10.1016/j.earlhumdev.2010.12.009 21262556

[B44] May P. A. Blankenship J. Marais A.-S. Gossage J. P. Kalberg W. O. Barnard R. (2014). Approaching the prevalence of the full spectrum of fetal alcohol spectrum disorders in a South. *Alcohol Clin. Exp. Res.* 37 818–830. 10.1111/acer.12033 PMC3610844 23241076

[B45] May P. A. Gossage J. P. Brooke L. E. Snell C. L. Marais A. S. Hendricks L. S. (2005). Maternal risk factors for fetal alcohol syndrome in the Western cape province of South Africa: a population-based study. *Am. J. Public Health* 95 1190–1199. 10.2105/ajph.2003.037093 15933241 PMC1361810

[B46] McLeod W. Brien J. Loomis C. Carmichael L. Probert C. Patrick J. (1983). Effect of maternal ethanol ingestion on fetal breathing movements, gross body movements, and heart rate at 37 to 40 weeks’ gestational age. *Am. J. Obstet. Gynecol.* 145 251–257. 10.1016/0002-9378(83)90501-x 6849360

[B47] Mulder E. J. H. Morssink L. P. Van Der Schee T. Visser G. H. A. (1998). Acute maternal alcohol consumption disrupts behavioral state organization in the near-term fetus. *Pediatr. Res.* 44 774–779. 10.1203/00006450-199811000-00022 9803461

[B48] Myers M. M. Elliott A. J. Odendaal H. J. Burd L. Angal J. Groenewald C. (2017). cardiorespiratory physiology in the safe passage study: protocol, methods and normative values in unexposed infants. *Acta Paediatr.* 106 1260–1272. 10.1111/apa.13873 28419567 PMC5530586

[B49] Oncken C. Kranzler H. Malley P. O. Gendreau P. Campbell W. A. (2002). The effect of cigarette smoking on fetal heart rate characteristics. *Obstet. Gynecol.* 99 751–755. 10.1016/s0029-7844(02)01948-8 11978283

[B50] Péterfi I. Kellényi L. Péterfi L. Szilágyi A. (2019). The short-term effect of smoking on fetal ECG. *J. Matern. Neonatal Med.* 32 724–733. 10.1080/14767058.2017.1390560 28992716

[B51] Phelan J. P. (1980). Diminished fetal reactivity with smoking. *Am. J. Obstet. Gynecol.* 136 230–233. 10.1016/0002-9378(80)90602-x 7352504

[B52] Pincus S. M. Cummins T. R. Haddad G. G. (1993). Heart rate control in normal and aborted-SIDS infants. *Am. J. Physiol.* 264 R638–R646. 8457020 10.1152/ajpregu.1993.264.3.R638

[B53] Pini N. Lucchini M. Esposito G. Tagliaferri S. Campanile M. Magenes G. (2021). A machine learning approach to monitor the emergence of late intrauterine growth restriction. *Front. Artif. Intell.* 4:622616. 10.3389/frai.2021.622616 33889841 PMC8057109

[B54] Pini N. Shuffrey L. C. Lucchini M. Sania A. Nelson M. E. Nugent J. D. (2019). “Cluster analysis of alcohol consumption during pregnancy in the safe passage study,” in *Proceedings of the 2019 41st Annual International Conference of the IEEE Engineering in Medicine and Biology Society (EMBC)*, Berlin, 1338–1341. 10.1109/EMBC.2019.8857428 31946140

[B55] Popova S. Lange S. Probst C. Parunashvili N. (2017). Prevalence of alcohol consumption during pregnancy and fetal alcohol spectrum disorders among the general and Aboriginal populations in Canada and the United States. *Eur. J. Med. Genet.* 60 32–48. 10.1016/j.ejmg.2016.09.010 27638329

[B56] Popova S. Lange S. Shield K. Mihic A. Chudley A. E. Mukherjee R. A. S. (2016). Comorbidity of fetal alcohol spectrum disorder: a systematic review and meta-analysis. *Lancet* 387 978–987. 10.1016/s0140-6736(15)01345-8 26777270

[B57] Quigley M. E. Sheehan K. L. Wilkes M. M. Yen S. S. C. (1979). Effects of maternal smoking on circulating catecholamine levels and fetal heart rates. *Am. J. Obstet. Gynecol.* 133 685–690. 10.1016/0002-9378(79)90019-x 426024

[B58] Reed T. E. (1978). Racial comparisons of alcohol metabolism: background, problems, and results. *Alcohol Clin. Exp. Res.* 2 83–87. 10.1111/j.1530-0277.1978.tb04699.x 345858

[B59] Reeves S. Bernstein I. (2008). Effects of maternal tobacco-smoke exposure on fetal growth and neonatal size. *Expert Rev. Obstet. Gynecol.* 3 719–730. 10.1586/17474108.3.6.719 19881889 PMC2770192

[B60] Rubertsson C. Börjesson K. Berglund A. Josefsson A. Sydsjö G. (2011). The Swedish validation of Edinburgh postnatal depression scale (EPDS) during pregnancy. *Nord. J. Psychiatry* 65 414–418. 10.3109/08039488.2011.590606 21728782

[B61] Rurak D. A. N. Lim K. E. N. Sanders A. R. I. Brain U. Riggs W. Oberlander T. I. M. F. (2011). Third trimester fetal heart rate and Doppler middle cerebral selective serotonin reuptake inhibitor exposure. *Pediatr. Res.* 70 96–101. 10.1203/pdr.0b013e31821ba11a 21436759

[B62] Sania A. Pini N. Nelson M. E. Myers M. M. Shuffrey L. C. Lucchini M. (2020). The K nearest neighbor algorithm for imputation of missing longitudinal prenatal alcohol data. *Res. Squ..* 10.21203/rs.3.rs-32456/v1 PMC11811783 39935524

[B63] Saul J. P. (1990). Beat-to-beat variations of heart rate reflect modulation of cardiac autonomic outflow. *Physiology* 5 32–37. 10.1152/physiologyonline.1990.5.1.32

[B64] Schneider U. Frank B. Fiedler A. Kaehler C. Hoyer D. Liehr M. (2008). Human fetal heart rate variability-characteristics of autonomic regulation in the third trimester of gestation Human fetal heart rate variability-characteristics of autonomic regulation in the third trimester of gestation ^∗^, ^∗∗^. *J. Perinat. Med.* 36 433–441. 18605969 10.1515/JPM.2008.059

[B65] Schoendorf K. C. Kiely J. L. (1992). Relationship of sudden infant death syndrome to maternal smoking during and after pregnancy. *Pediatrics* 90 905–908. 1437432

[B66] Scragg R. Mitchell E. A. Taylor B. J. Stewart A. W. Ford R. P. Thompson J. M. (1993). Bed sharing, smoking, and alcohol in the sudden infant death syndrome. New Zealand Cot death study group. *BMJ* 307 1312–1318. 10.1136/bmj.307.6915.1312 8257885 PMC1679405

[B67] Shuffrey L. C. Fifer W. P. (2020). “Prenatal risk factors and neurodevelopment,” in *Encyclopedia of Infant and Early Childhood Development*, ed. Benson J. B. B. T. (Oxford: Elsevier), 608–620. 10.1016/b978-0-12-809324-5.23054-x

[B68] Shuffrey L. C. Myers M. M. Isler J. R. Lucchini M. Sania A. Pini N. (2020). Association Between prenatal exposure to alcohol and tobacco and neonatal brain activity: results from the safe passage study. *JAMA Netw. Open* 3:e204714. 10.1001/jamanetworkopen.2020.4714 32396193 PMC7218492

[B69] Shuffrey L. C. Myers M. M. Odendaal H. J. Elliott A. J. du Plessis C. Groenewald C. (2019). Fetal heart rate, heart rate variability, and heart rate/movement coupling in the safe passage study. *J. Perinatol.* 39 608–618. 10.1038/s41372-019-0342-9 30833637 PMC6483837

[B70] Silva P. D. Miller K. D. Madden J. Keegan K. A. (1987). Abnormal fetal heart rate pattern associated with severe intrapartum maternal ethanol intoxication. A case report. *J. Reprod. Med.* 32 144–146. 3560078

[B71] Slotkin T. A. (1998). Fetal nicotine or cocaine exposure: which one is worse? *J. Pharmacol. Exp. Ther.* 285 931–945. 9618392

[B72] Spyridou K. Chouvarda I. Hadjileontiadis L. Maglaveras N. (2017). The effect of cigarette smoking on fetal heart rate tracing during pregnancy. *J. Perinat. Med.* 45 403–411. 10.1515/jpm-2015-0275 27054592

[B73] Vardavas C. I. Chatzi L. Patelarou E. Plana E. (2010). Smoking and smoking cessation during early pregnancy and its effect on adverse pregnancy outcomes and fetal growth. *Eur. J. Pediatr.* 169 741–748. 10.1007/s00431-009-1107-9 19953266

[B74] Voegtline K. M. Costigan K. A. Henderson J. L. DiPietro J. A. (2016). Fetal heart rate and motor development in overweight and obese pregnant women. *Int. J. Gynecol. Obstet.* 133 103–107. 10.1016/j.ijgo.2015.08.006 26797193 PMC4808629

[B75] Wisborg K. Kesmodel U. Henriksen T. B. Olsen S. F. Secher N. J. (2001). Exposure to tobacco smoke in utero and the risk of stillbirth and death in the first year of life. *Am. J. Epidemiol.* 154 322–327. 10.1093/aje/154.4.322 11495855

[B76] Zeskind P. S. Gingras J. L. (2018). Maternal cigarette-smoking during pregnancy disrupts rhythms in fetal heart rate. *J. Pediatr. Psychol.* 31 5–14. 10.1093/jpepsy/jsj031 15905420

