# Supplementary figures and images for: Effects of Prenatal Exposure to Alcohol and Smoking on Fetal Heart Rate and Movement Regulation
Source: Front Physiol. 2021 Jul 30;12:594605. doi: 10.3389/fphys.2021.594605 (PMC8363599; doi:10.3389/fphys.2021.594605)

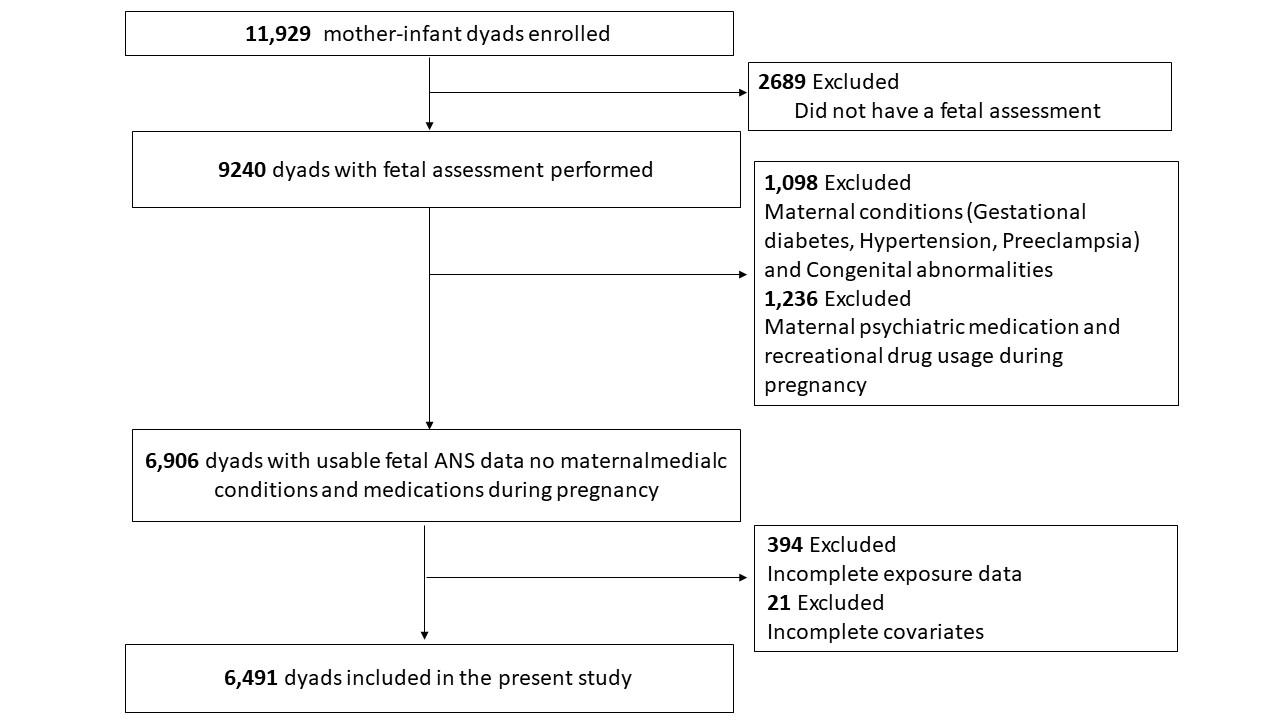

Supplement: Supplementary Figure 1 — Study flowchart. [file Image_1.JPEG]
