# Supplementary material for: Effects of Prenatal Exposure to Alcohol and Smoking on Fetal Heart Rate and Movement Regulation
Source: Front Physiol. 2021 Jul 30;12:594605. doi: 10.3389/fphys.2021.594605 (PMC8363599; doi:10.3389/fphys.2021.594605)
Supplement: Supplementary Material 1 — Missing data imputation. [file Data_Sheet_1.docx]

***S. M. 1 Missing Data Imputation.***

All pregnant women in the Safe Passage Study provided information on daily drinking and smoking for the last reported drinking day and 30 days prior. For estimates of alcohol use, out of 3.2 million person-days of observation, data were missing for 0.36 million (11.4%) days. For estimates of smoking, out of 0.47 million person-weeks, data were missing for 0.13 person-weeks (27.7%).

We imputed missing exposure data using a machine learning algorithm; “K Nearest Neighbor” (K-NN). K-NN imputes missing values for a participant using data of other participants closest to it. Since participants with no missing days may not be comparable to those with missing data, segments from those with complete and incomplete data were included as a reference. Imputed values were weighted for the distances from nearest neighbors and matched for day of week.

Validation analyses showed we classified drinking status accurately 64 % of validation segments. For 31% of the segments, imputed values were within +/-1 drink/day of the actual. We classified smoking status correctly in 93% of randomly deleted segments, and, for 86% segments imputed data were within +/-1 cigarette/day of the actual.

Subjects with more than 200 days of missing drinking data and subjects who did not have any drinking data in first trimester was excluded from the analyses. Subjects who did not have smoking data for at least one visit was excluded from imputation. These data which we consider unimputable were not included in clustering and therefore are not part of this paper.

***S. M. 2 Alcohol and Smoking Cluster Analysis.***

To distinguish the effect of different patterns of PAE and PTE during pregnancy on fetal autonomic control, we implemented an unsupervised data-driven clustering technique for the characterization of maternal drinking and smoking longitudinal profiles.

The cluster analysis of alcohol consumption during pregnancy in the Safe Passage Study has been previously published [1]. In summary, the R-package clValid [2] was utilized to compare different clustering methodologies. We determined the most appropriate probability density functions and number of clusters based on several figures of merit, such as connectivity measures, the Dunn index, and silhouette measures. Finite mixture models were selected and implemented using the R-package mclust [3] from derived daily alcohol consumption data for 10,279 participants in the Safe Passage Study. Six features of alcohol consumption were utilized to the cluster analysis: the sum of standard drinks per day in trimesters 1, 2, and 3 and the count of binge drinking events (≥ 4 drinks on a given day) in trimesters 1, 2, and 3. The model identified ten alcohol trajectory groups, with one group consisting of non-drinkers (n=5,915).

To identify patters of smoking exposure, hierarchical clustering was implemented using the R-packages hclust [4] and agnes [2] for 10,941 participants with available smoking information. Four features were used in the model: average cigarettes smoked per week in trimesters 1, 2, and 3 and a quit smoking variable, defined as < 1 average cigarettes per week in trimesters 2 and 3. We utilized Euclidean distance and the linkage method was Ward’s minimum variance method. Women with a total trimester-level average cigarette consumption <1 had that corresponding value set to zero. For the remaining 5,155 participants, any outliers for each feature were assigned with a value equal to the Mean + (4*SD). Additionally, features were z-scored to obtain a zero-mean and unitary-variance. The model identified five smoking trajectory groups, with one group consisting of non-smokers (n=5,786). For some alcohol and smoking exposure cluster cross-tabulations, there were small N’s which also had fetal HRV data. Therefore, in the present analysis we merged cluster groups to create six categories of PAE (no alcohol, low quit early, high quit early, low continuous, moderate continuous, high continuous) and a four-level PTE variable (no, quit early, low continuous, moderate/high continuous).

***S. M. 3 Data Processing.***

With custom MATLAB programs, the fetal HR signal was processed to detect signal loss and artifact, generally due to fetal and/or maternal movement or repositioning of the transducer. Fetal HR values < 100 or > 200 bpm were set to missing and substituted via linear interpolation. The resultant fetal HR series was then low–pass filtered at 3 Hz using a 16–point finite impulse response filter. Artifacts in the filtered fetal HR were further identified when the absolute sample–to–sample change in FHR exceeded 5 bpm and substituted by linear interpolation. The resulting signal was then analyzed in 60 second epochs. Epochs were excluded from analyses if 1) FHR signal loss for a gap was > 5 seconds, or 2) cumulative excluded data was > 20 seconds (not including gaps of 2 seconds or less), or 3) if the epoch had > 30% excluded data.

Fetal movement data was calibrated for each collection system by determining the minimum and maximum values analog-to-digital units (AD units) across all studies collected on that system and then transforming the signal to an arbitrary 0 to 100 scale [5]. Only fetuses with at least 4 minutes of usable data were included in the subsequent analysis.

***S. M. 4 Fetal State Coding***.

In the literature there are several studies addressing the use of fetal HR patterns, body and eye movements to code fetal behavioral states [6]. However, in this study we did not have synchronous ultrasound to visualize fetal body or eye movements. Previous reports have suggested that in the last weeks of gestation coincidence among all three parameters is sufficiently high to provide behavioral states determination from HR patterns alone [7], [8]. Therefore, fetal state coding was accomplished by highly trained coders through visual inspection of the FHR tracings only.

State 1F, also known as the quiet fetal behavioral state, was coded when fetal HR exhibited flat tracings (i.e. narrow oscillation bandwidth) with few and small accelerations. State 2F, also known as the active fetal behavioral sleep state, was coded when fetal HR tracings displayed more frequent and larger accelerations than in state 1F and with periods between accelerations displaying a greater oscillation band width than state 1F. State 3F, also known as the quiet awake fetal behavioral sleep state, exhibited tracings similar to State 1F in that there are few accelerations, but with bandwidths of oscillations broader than 1F and more similar to the bandwidth between accelerations of 2F. State 4F, also known as the active awake fetal behavioral state, was coded when fetal HR large and prolonged accelerations often fused into sustained tachycardia were present. The percentage of time spent in 3F and 4F during pregnancy is relatively small, thus our analysis focused on data from 1F and 2F.

**References**

[1] N. Pini *et al.*, “Cluster Analysis of Alcohol Consumption during Pregnancy in the Safe Passage Study,” in *2019 41st Annual International Conference of the IEEE Engineering in Medicine and Biology Society (EMBC)*, 2019, pp. 1338–1341.

[2] G. Brock, V. Pihur, S. Datta, and S. Datta, “clValid, an R package for cluster validation,” *J. Stat. Softw. (Brock al., March 2008)*, 2011.

[3] L. Scrucca, M. Fop, T. B. Murphy, and A. E. Raftery, “mclust 5: Clustering, Classification and Density Estimation Using Gaussian Finite Mixture Models.,” *R J.*, vol. 8, no. 1, pp. 289–317, Aug. 2016.

[4] P. Langfelder and S. Horvath, “Fast R functions for robust correlations and hierarchical clustering,” *J. Stat. Softw.*, vol. 46, no. 11, 2012.

[5] J. A. DiPietro, K. A. Costigan, and E. K. Pressman, “Fetal movement detection: Comparison of the Toitu actograph with ultrasound from 20 weeks gestation,” *J. Matern. Med.*, vol. 8, no. 6, pp. 237–242, 1999.

[6] J. G. Nijhuis, H. F. R. Prechtl, C. B. Martin, and R. S. G. M. Bots, “Are there behavioural states in the human fetus?,” *Early Hum. Dev.*, vol. 6, no. 2, pp. 177–195, 1982.

[7] M. Pillai and D. James, “Behavioural states in normal mature human fetuses,” *Arch. Dis. Child.*, vol. 65, no. 1 SPEC NO, pp. 39–43, 1990.

[8] G. H. A. Visser, E. J. H. Mulder, H. Stevens, and R. Verweij, “Heart rate variation during fetal behavioural states 1 and 2,” *Early Hum. Dev.*, vol. 34, no. 1–2, pp. 21–28, 1993.
